# Supplementary material for: Pairing statistics and melting of random DNA oligomers: Finding your partner in superdiverse environments
Source: PLoS Comput Biol. 2022 Apr 11;18(4):e1010051. doi: 10.1371/journal.pcbi.1010051 (PMC9022813; doi:10.1371/journal.pcbi.1010051)
Supplement: S1 Text — Evidence of Out-of-Equilibrium Conditions; Perfect pairing probability with different energies and number of nucleobases types. Fig A: Evidence of out-of-equilibrium behavior for 12N. T dependence of the fraction of duplexed strands θe measured while heating and cooling at 1°C/min. Fig B: Evidence of out-of-equilibrium behavior for 20N. Absorbance vs. T measured upon heating after one month equilibration at 4°C. Fig C: θA*B*, fraction of paired 12A* − 12B* in 12N, determined from the model and via CQ experiments with different cooling rates. Fig D: θ0(fCG) computed with different values of fCG in 8N. Fig E: θ0(nb) computed with different values of nb in 12N. (PDF) [file pcbi.1010051.s001.pdf]

S1 Text. Further Results
Pairing Statistics and Melting of Random DNA Oligomers: finding your Partner in Superdiverse Environments

I. EVIDENCE OF OUT-OF-EQUILIBRIUM CONDITIONS

Data presented in the main text have been acquired in equilibrated samples. The equilibrium condition is not trivial, since the lifetime of DNA duplexes dramatically depends on the pairing energies, and thus on the length of the oligomers, a condition relevant for 20N. A relevant role is also played by DNA concentration, since it hampers molecular self-diffusion. We report here evidence of out-of-equilibrium conditions for 12N at high rsDNA concentration (c_{rsDNA} = 25 g/l) and for 20N at low rsDNA concentration (c_{rsDNA}= 0.04g/l) when heating and cooling rates are not sufficiently small. The out-of-equilibrium behavior of rsDNA will be the topic of a forthcoming work.

A. Melting Experiments

Melting experiments for 12N at c_{rsDNA} = 25 g/l and c_{NaCl} = 450 mM, with cooling and heating rates of $1°C/min$, show a significant difference between heating and cooling ramps (red and blue dots in Fig A, respectively), which are about $4°C$ apart over the T range 20-40$°C$. Moreover, both heating and cooling θ_e occur at markedly lower T compared to the the theoretical prediction (dashed line). Both observations indicate that this sample has not reached equilibrium and that at this experimental cooling rate rsDNA forms duplexes that contain more defects than in the theoretical prediction, and thus melt at a lower T. The better stability observed in heating experiments also points to the fact that samples which have had a longer residence time at low T, manage to form duplexes with partially improved pairing. Analogous behavior has been observed at c_{NaCl} = 0.15M and 1.0 M.

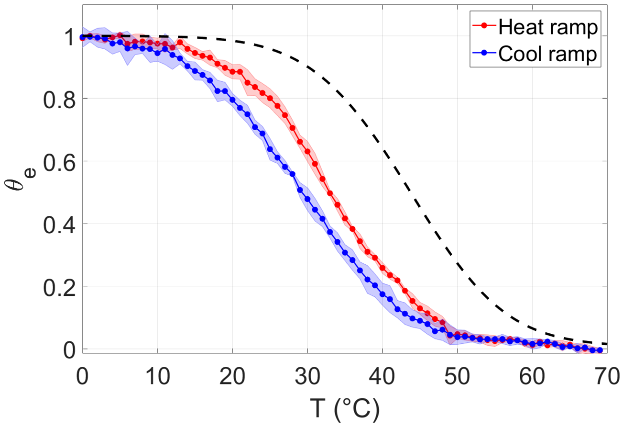

Fig A. Evidence of out-of-equilibrium behavior for 12N rsDNA at c_{12N} = 25 g/l and c_{NaCl} = 450 mM. T dependence of the fraction of duplexed strands θ_e measured while heating (red dots) and cooling (blue dots) at $1°C/min$. Dashed line: predicted melting curve for the same conditions.

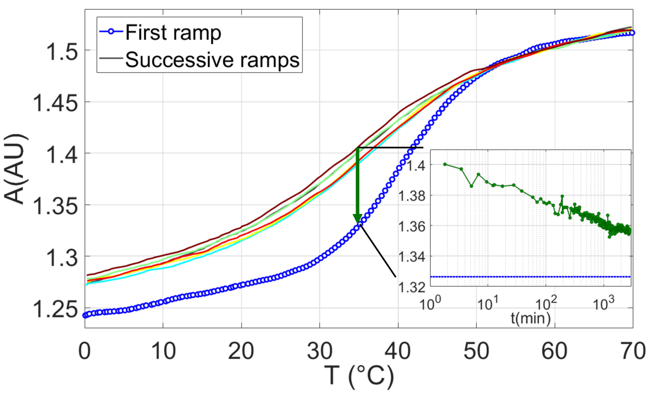

Fig B. Evidence of out-of-equilibrium behavior for 20N at c_{rsDNA} = 0.04 g/l and c_{NaCl} = 450mM. Blue dots: absorbance vs. T measured upon heating after one month equilibration at $4°C$. Successive heat and cool ramps (colored lines) clearly markedly differ from the first ramp in both amplitude and T_m. Inset: time dependence of the absorbance after quenching from from T = $80°C$ to T = $35°C$ from T = $80°C$.

θ_e reported in the main text for 20N refers to the first heating ramp of samples which were held at 4^oC for several days. Fig B shows the such first heating ramp absorbance data A(T) (blue dots) together with $A(T)$ measured in the successive $1°C/min$ cooling and heating ramps (colored lines) for 20N, c_{rsDNA} = 0.04 g/l and c_{NaCl} = 450 mM. Differences both in the absorbance at low T and in the T dependence are evident. The smaller A(T) of the first ramp at low T indicates a lower average molar extinction coefficients ϵ, in turn reflecting the different quality pairing of DNA duplexes. In fact, less defected duplexes have higher hypocromicity and consequently lower ϵ and A(T) (see Eq. 1 in SI 2 Materials and Methods). The inset shows the recovery of absorbance after having quenched the sample from high T at T = $35°C$. The time needed to recover the value A(T = $35°C$) measured during the first heating ramp (dashed blue line) is longer than 48 hours. Similar behavior is observed for 20N with c_{NaCl} = 0.15 and 1M.

B. Contact-Quenching Experiments

The out-of-equilibrium behaviour of 12N melting at $c_{rsDNA} = 25g/l$ is also apparent in CQ experiments. In Fig C, we present $\theta_{A^*B^*}$, the fraction of paired $12A^* - 12B^*$ in 12N at $c_{rsDNA} = 25g/l$ (i.e. $\phi = 200$), measured with two different cooling rates. As apparent, $\theta_{A^*B^*}$ saturates at a larger value when the cooling rate is slower (blue dots), demonstrating that the intrinsic dynamics of the system is not faster than the experimental times, as it should to grant equilibration. The difference between the curves indicates that, at this concentration, the unbinding rates of $12A^*$ and $12B^*$ with rsDNA oligomers slows their exploration of the conformation space to times larger than those allowed by the cooling ramps here considered. In these conditions, as in Fig A, the agreement with theoretical predictions (dashed red line in Fig C) is, as expected, quite poor.

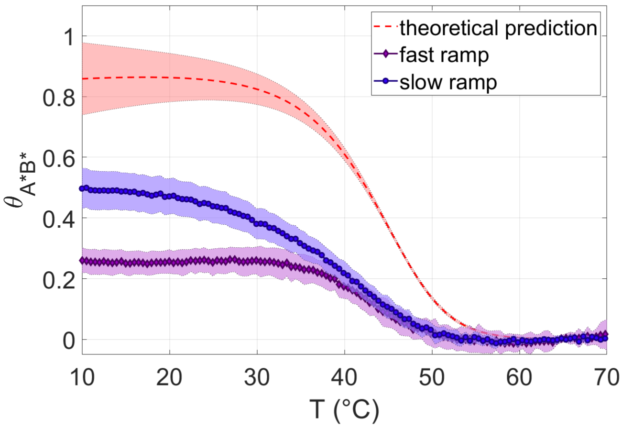

Fig C. $\theta_{A^*B^*}$, fraction of paired $12A^* - 12B^*$ in 12N, determined from the model (red dashed lines) and via CQ experiments with different cooling rates: $1°C/min$ (purple diamonds) and with $0.2°C/min$ (blue dots). The stoichiometric ratio is $\phi = 2 \cdot 10^2$ (i.e. $c_{rsDNA} = 25g/l$). $c_{NaCl} = 0.15M$. Both experimental curves are obtained as an average of 5 cooling ramps.

II. PERFECT PAIRING PROBABILITY WITH DIFFERENT ENERGIES AND NUMBER OF NUCLEOBASES TYPES

In Fig. 6(a) of the main text, we have shown the fraction of duplexes $\theta_{\boldsymbol{\alpha}}$ for the most probable pairing qualities $\boldsymbol{\alpha}$, after averaging on f_{CG}. Here we show, in Fig D, the predicted fraction of perfect duplexes $\theta_{\mathbf{0}}^{(f_{CG})}$ detailed for various CG content. As apparent, $\theta_{\mathbf{0}}^{(f_{CG})}$ significantly depends on the CG fraction, since a larger binding energy increases both the thermal stability, which varies of about $40°C$ between the two extreme situations, and the value at low T, which ranges from 8% to 26%. It should however be noticed that, even for $f_{CG} = 1$ the probability of perfect pairing in rsDNA solutions is well below 1.

We have so far considered natural nucleic acids, in which the number of types of bases is $n_b = 4$. To explore the relevance of degeneracy, it is easy to extend the evaluation of $\theta_{\mathbf{0}}^{(f_{CG})}$ to artificial systems with a generic value of n_b, using the averaged pairing free energies, as for $f_{CG} = 1/2$. Indeed, considering generic values for n_b affects the pairing statistics of the rsDNA only the through the degeneracy of the pairings, which can be better appreciated by re-writing Eq. 5 of the main text making the dependence on n_b explicit:

$$g(L, n_b, \boldsymbol{\alpha}) = n_b^{|\alpha_s|} (n_b - 1)^{(\alpha_{e1}+\alpha_{e2}+\alpha_i)} \cdot \cdot \binom{L - 2 - |\alpha_s| - \alpha_{e1} - \alpha_{e2}}{\alpha_i}. \tag{1}$$

Of course, when computing the melting curves, normalization involves the quantity n_b^L, instead of 4^L.

As apparent in Fig E, $\theta_{\mathbf{0}}$ is maximum for minimum n_b. However, even for $n_b = 2$ the fraction of perfect pairs is still far from 1, further strengthening the key role of molecular diversity in the pairing statistics.

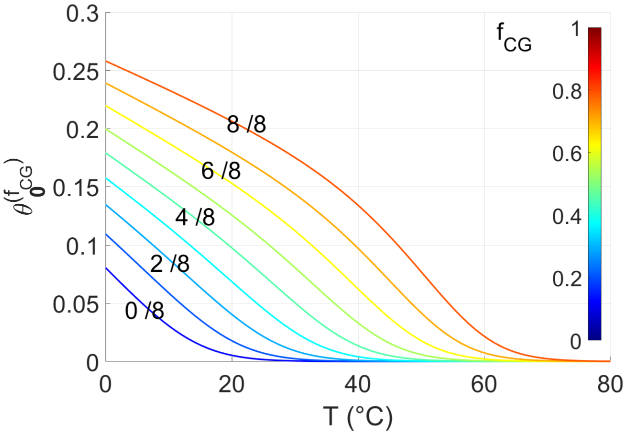

Fig D. $\theta_{\mathbf{0}}^{(f_{CG})}$ computed with different values of f_{CG} in 8N, at a concentration of $25g/l$ and NaCl 1M.

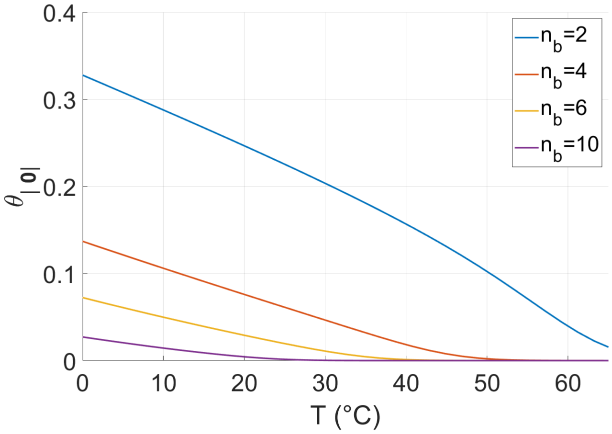

Fig E. $\theta_{\mathbf{0}}^{(n_b)}$ computed with different values of n_b in 12N, at a concentration of $25g/l$ and NaCl 1M.
