# Supplementary material for: Pairing statistics and melting of random DNA oligomers: Finding your partner in superdiverse environments
Source: PLoS Comput Biol. 2022 Apr 11;18(4):e1010051. doi: 10.1371/journal.pcbi.1010051 (PMC9022813; doi:10.1371/journal.pcbi.1010051)
Supplement: S2 Text — Characterization of rsDNA synthesis; Measurement of rsDNA concentration; UV Absorbance: Experimental Setup; Analysis of UV Absorbance Data; Characterization of A* and B* Fluorescence; Contact-Quenching Data Analysis; Free Energy of A*B* Duplex. Fig A: HPLC traces of 12N compared with two different 12mers. HPLC traces of 8N, 12N and 20N. Fig B: Quartz microfluidic. Quantum Northwest Peltier. Fig C: Temperature calibration of T measured by a thermistor in contact with the microfluidic cell vs. the internal control Tpeltier. Fig D: Steps in the analysis of absorbance data A(T) of 12N used to extract the melting curves θe. Fig E: Fluorescence Emission Spectra of labeled DNA systems: A*, B*, A*B*, A*B and AB*. Fig F: Absorbance Spectra of labeled DNA systems: A*, B*, A*B*, A*B and AB*. Fig G: Simplified representation of the 12A*B* duplex, showing the relative size of DNA duplex, linker and fluorescent moieties FAM and TexasRed. Fig H: fluorescence intensity of TexasRed vs. T in a solution of 12A* + 12B*. Fig I: Normalized fluorescence intensity of TexasRed in a solution of 12A*, 12B* and 12B. Fig J: Average normalized fluorescence of 8A*+8B*. The fit enables determining the linear drift at low temperatures, corresponding to the signal of fully paired A*B*. Fig K: Tm vs A*B* concentration measured by CQ for the following systems. (PDF) [file pcbi.1010051.s002.pdf]

S2 Text. Materials and Methods
Pairing Statistics and Melting of Random DNA Oligomers: finding your Partner in Superdiverse Environments

I. CHARACTERIZATION OF RSDNA SYNTHESIS

The solid-phase synthesis of 20N rsDNA was previsously characterized by MALDI-TOF, as described in the S.I. of Ref. [1], where we found that the width of the mass distribution was compatible with the expectation for a random sequence system.

In the context of this work, we also characterized the rsDNA oligomers by HPLC. HPLC experiments were performed at $T = 40°C$ on DNA solution prepared at $c_{DNA} = 0.1g/l$. In Fig A(a) we show HPLC traces of 12N compared with single strand oligomer 12ss (CTATGCCACCTA) and self-complementary 12-mer DD (CGCGAATTCGCG). The main HPLC peaks of 12ss and DD are apart much more than their width, and occurs within the broader range of the 12N peak profile. The broadness of the 12N peak is the signature of a variety of DNA sequences eluted at slightly different times due to both their different A,T,C and G contents and relative order. Also to be noticed that the center of the 12N trace coincides with the main peak of 12 ss, which has $f_{CG} = 0.5$. In Fig A(b) we show HPLC traces of the three rsDNA oligomers used in this work, 8N, 12N and 20N. All HPLC traces share the same shape, with their centers occurring at higher times according to their length, as expected.

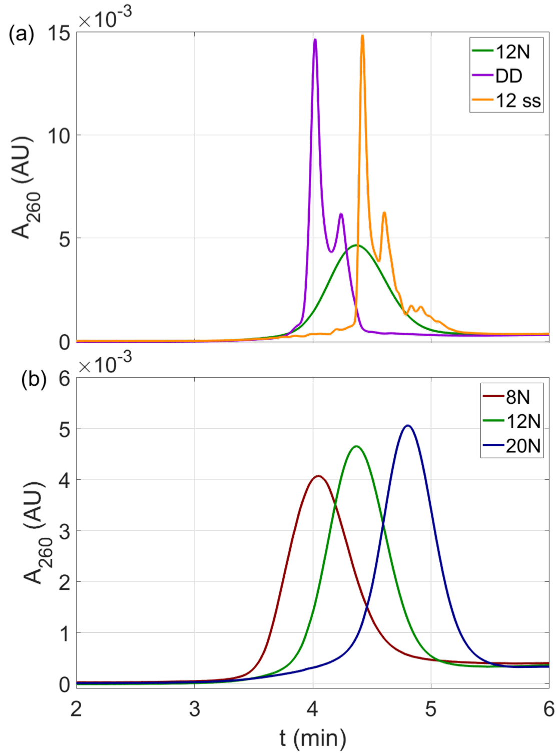

Fig A. (a) HPLC traces of 12N compared with two different 12mers, 12ss (CTATGCCACCTA) and DD(CGCGAATTCGCG). (b) HPLC traces of 8N, 12N and 20N. HPLC experiments were performed at $T = 40°C$ and DNA solutions were prepared at c_{DNA}= 0.1g/l.

Although HPLC and MALDI do not demonstrate the actual full randomness of the rsDNA synthesis, they clearly support the notion that the distribution of sequences if broad. Indeed, for the results and observations of this study to hold it is not necessary that the randomness is perfect, but rather that the distributions of defects are the same as in a random system, a condition that could be fulfilled even in a system with imperfect - but still broad - randomness.

It is interesting to compare the number of distinct molecular species in the rsDNA solutions with the actual number of molecules in the experimental samples. Among the system considered, the most extreme diversity is that of 20N, where the number of sequences is $4^{20} \approx 1.1 \cdot 10^{12}$. Among the samples used in the experiments, the ones with the smallest number of total molecules are those used in melting experiments with the $1\,cm$ cuvette, in which we use $1\,mL$ of solution at a concentration of $c = 0,04g/l \approx 6mM$. In this case the total number of molecules in the sample is $N \approx 4 \cdot 10^{16}$, thus granting $\approx 10^4$ replicas per molecular type.

II. MEASUREMENT OF RSDNA CONCENTRATION

The absorbance A of a DNA solution is proportional to the oligomers concentration and the path length [2]:

$$A = \epsilon\, l\, c_{DNA} \tag{1}$$

where l is the optical path length, c_{DNA} is the DNA molar concentration and ϵ is the molar extinction coefficient of the DNA in the sample. The extinction coefficient for a particular sequence i is computed, according to the NN model [2], with the following expression:

$$\epsilon_i = \epsilon_i(ext_1) + \epsilon_i(ext_2) + \sum_{q=1}^{L-1} \epsilon_i(q) \tag{2}$$

where $\epsilon_i(ext_1)$ and $\epsilon_i(ext_2)$ represents the contribution of the first and the last bases of the DNA sequence and $\epsilon_i(q)$ are the extinction coefficients of the couples of bases in the position q and $q+1$ along the sequence i.

As introduced in the theoretical section, we will consider mixture of rsDNA oligomer where all possible sequences of length L are equally populated; therefore we need the average of the extinction coefficient $\langle\epsilon_i\rangle_i$ over all possible sequences in the rsDNA system. For rsDNA oligomers with generic length L we get:

$$\begin{aligned}\epsilon_{rsDNA,L} &= \langle\epsilon_i(ext_1)\rangle_i + \langle\epsilon_i(ext_2)\rangle_i + \sum_{q=1}^{L-1}\langle\epsilon_i(q)\rangle_i \\ &= 2\bar{\epsilon}_{ext} + (L-1)\bar{\epsilon}_{int}, \qquad (3)\end{aligned}$$

where $\bar{\epsilon}_{ext} = \langle\epsilon_i(ext_1)\rangle_i = \langle\epsilon_i(ext_2)\rangle_i = 5375(cm\,M)^{-1}$ and the internal term is $\bar{\epsilon}_{int} = \langle\epsilon_i(q)\rangle_i = 9572(cm\,M)^{-1}$ [2]. Consequently, for the rsDNA system of length L=12, we get $\epsilon_{12N} = 116041(cm\,M)^{-1}$. We perform the concentration characterization in melted condition at high temperatures, in order to avoid the hypocromicity term that is unknown in the case of rsDNA hybridization involving pairing errors. By UV absorbance we set c_{DNA} in the experimental samples.

III. UV ABSORBANCE: EXPERIMENTAL SETUP

To perform melting experiments at high DNA concentration two technological upgrades were necessary: 1) the quartz microfluidic cell shown in Fig B with path length l = 10 μm, which allowed us to increase c_{DNA} up to 20 - 30g/l; 2) Quantum Northwest Peltier hot/cold stage (shown in Fig B) combined with a metal (”T” shaped) cell holder which allowed us to explore a wider range of temperature T = 0-90 °C. Indeed, accessing to the lowest temperature range is crucial for the analysis of most melting curves, as explained below. Evaparotion was prevented by sealing the two noozles of cell with EPDM corks.

Because of the complex new cell holder and of the need of accurate T measurements, we carefully calibrated the thermostatic system. With repeated heating and cooling ramps in the interval -6°C to 100°C at 1°C/min, we measured T with a thermistor in contact with the microfluidic cell as a function of the inner Peltier probe temperature, $T_{peltier}$, shown in Fig B.

Melting experiments for diluted DNA solution ($c_{DNA} = 0.04g/l$) were performed using standard quartz cuvette hosted in the same call holder. T was in this case measured directly by a the thermistor inside the DNA solution. In diluted melting experiments, solutions in cuvette were covered by mineral oil in order to prevent evaporation. In all experiments a nitrogen constant flux was used to prevent condensation on both cuvette and microfluidic cell at low T.

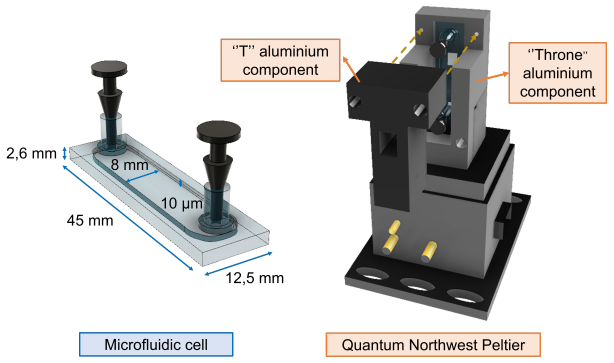

Fig B. Quartz microfluidic cell with path length l = 10μm sealed with black EPDM corks. Quantum Northwest Peltier coupled to a cell holder formed by the combination of two aluminum components, separately indicated.

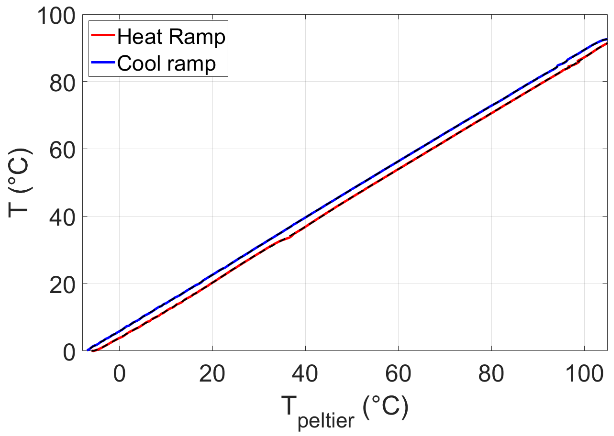

Fig C. Temperature calibration of T measured by a thermistor in contact with the microfluidic cell vs. the internal control $T_{peltier}$. Red and blue lines: calibration upon heating and cooling, respectively. Dashed line: polynomial fit.

IV. ANALYSIS OF UV ABSORBANCE DATA

To analyze and perform fitting on the absorbance data $A(T)$, we first applied a Savitzky-Golay smoothing filter (sgolayfilt Matlab function) with polynomial order 3. Fig Da shows such curves corresponding to serveral heating and cooling ramps performed consecutively on the same rsDNA solution. It is evident that no evaporation occurs and experiments are reproducible. Secondly, each smoothed $A(T)$ is fitted following standard protocols [3] in order to remove the contribution of the absorbance drift at high temperature $A_{HT}(T)$ and at low temperature $A_{LT}(T)$ so to extract the fraction of rsDNA single strands θ:

$$\theta(T) = \frac{A(T) - A_{LT}(T)}{A_{HT}(T) - A_{LT}(T)}. \qquad (4)$$

Although our aim is removing the high and low T drifts, in order to perform the fit necessary to determine

them we need to adopts a specific functional shape for θ, which we chose as:

$$\theta(T) = \frac{a_1}{1+e^{-a_2(T-a_3)}} \tag{5}$$

and A_{HT}, A_{LT} are described by:

$$\begin{aligned} A_{LT}(T) &= a_4 + a_5 T \\ A_{HT}(T) &= a_6 + a_7 T \end{aligned} \tag{6}$$

the experimental absorbance data A(T) are fitted by:

$$y(T) = \frac{a_1}{1+e^{-a_2(T-a_3)}}(a_6 - a_4 + (a_7 - a_5)T) + a_4 + a_5 T \tag{7}$$

where a_i, with $i = \{1, 7\}$, are free parameters.

Fig D(b) shows one measured A(T) together with the fitting line from Eq.(7) (dashed line) and the high and low T drifts A_{HT} and A_{LT} (blue line). Since we are interested in the fraction of paired rsDNA duplexes θ_e, we use the fit to determine A_{HT} and A_{LT}, remove then from the data and extract θ_e as:

$$\theta_e(T) = 1 - \theta(T) \tag{8}$$

Fig D(c) shows the experimental melting curves $\theta_e(T)$ obtained from the data in Fig D(a) after the removal of the drifts. Finally, these normalized $\theta_e(T)$ melting curves are averaged out. The resulting $\theta_e(T)$ is shown in Fig Dd (blue dots) with experimental uncertainty marked by light blue shading. The melting temperature T_m of the measurement is set by the condition $\theta_e(T = T_m) = 1/2$. Fig D(b) reveals how crucial it is to access the range of temperature 0-15°C to determine the absorbance drift in systems in which T_m is not large. For example, in the case here determined ($T_m \approx 30°C$) the melting of duplexes spreads down to below 15°C.

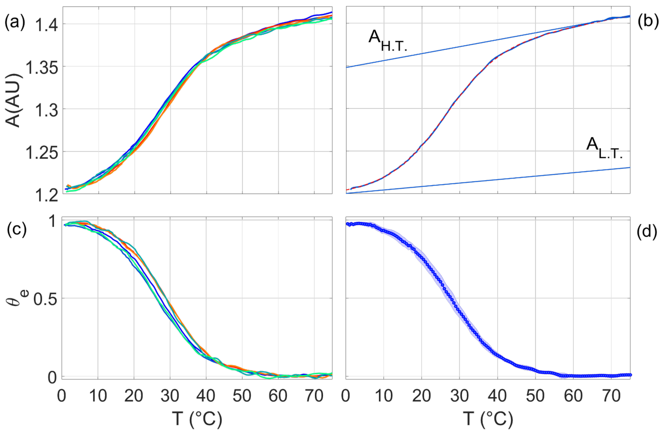

Fig D. Step in the analysis of absorbance data $A(T)$ of 12N ($c_{rsDNA} = 0.04$ g/l, $c_{NaCl} = 1$M) used to extract the melting curves θ_e. (a) $A(T)$ measured in consecutive heating and cooling ramps, after smoothing. (b) $A(T)$ (blue line) fitted with Eq. (7) (dashed red line). Blue lines: linear fit at the high and low T drift as in Eq. (6). (c) θ_e curves obtained after drift subtraction as in Eq. (4). (d) θ_e obtained as an average over the curves in panel (c). Experimental uncertainty is shown as the shaded light blue area around the data point.

V. CHARACTERIZATION OF A* AND B* FLUORESCENCE

Here we describe the florescence signal that enabled to detect the hybridizing of DNA strands labeled with the fluorophores Fam and TexasRed, and discuss why we identify it as a Contact-Mediated Quenching. We studied the absorption and fluorescence emission spectra of Fam and TexasRed linked to the complementary DNA strands A and B, respectively, in solutions having different compositions: A*, B* , A*+B* , A*+B and A+B*, where the asterisk marks the presence of thre fluorescent tag on the molecule. Fig E shows the fluorescence intensity I_f measured with a fluorimeter in the range from $\lambda = 470nm$ to $\lambda = 700nm$, with excitions at $\lambda_{FAM} = 492nm$ and $\lambda_{TexasRed} = 593nm$. We find the emission peak of the two fluorophores were expected: at $\lambda \approx 520nm$ for FAM and $\lambda \approx 610nm$ for TexasRed. Although the two florophores would be suitable for FRET, with TexasRed emission following Fam excitation, here the dominant effect is the quenching of both florophores upon forming the A*B* duplex. Indeed, Fig E shows that: i) the pairing of fluorophore-conjugated strands with untagged strands, leaves the fluorescent emission unchanged, as apparent comparing the emission from the A* and A*B, and from the B* and AB* solutions; ii) the formation of A*B* duplexes has a dramatic effect on I_f of both fluorophores. Upon A*B* duplexing, the emission of TexasRed, exited at $\lambda_{TexasRed} = 593nm$, is reduced to about half of the signal of A* or A*B. Analogously, the emission wavelengths of FAM, excited at $\lambda_{FAM} = 492nm$, is also approximately reduced to half of the signal of B* or AB*. We exploit this marked change in I_f to obtain information on the formation of A*B* duplexes when immersed in the sea of competitive interactions of rsDNA.

To gain understanding in the nature of the phenomenon, we measured the absorption spectrum of these systems. In Fig F we can appreciate that A*B* duplexing partially modifies the absorbance of both florophores, but not enough to justify the drop in emission described above. In particular, the A*B* absorbance is higher than B* and AB*.

A useful clue for the explanation of the A*B* fluorescence quenching can be found when considering the sizes of the involved molecules: in Fig G we show a schematic representation of the A*B* duplex which however respects the realtive size of double helix and fluorescent moieties. The size of the flexible linkers between fluorophores and DNA strands easily enables both the contact of FAM and TexasRed once the double helix is formed and their flat stacking on the blunt duplex terminal. The structure of A*B* is thus compatible

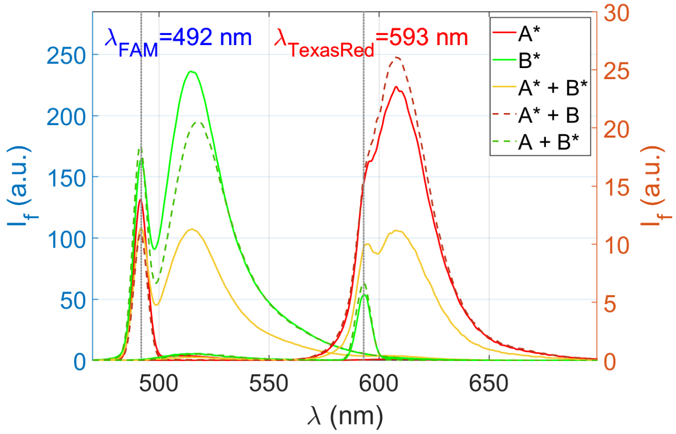

Fig E. Fluorescence Emission Spectra of labeled DNA systems: A* (red line), B* (green line), A*B* (yellow line), A*B (dark-red dashed-line) and AB* (dark-green dashed-line). Left y axis: FAM fluorescent intensity I_f, upon excitation at $\lambda_{FAM} = 492nm$. Right y axis: fluorescent intensity I_f of Texasred, upon excitation at $\lambda_{TexasRed} = 593nm$.

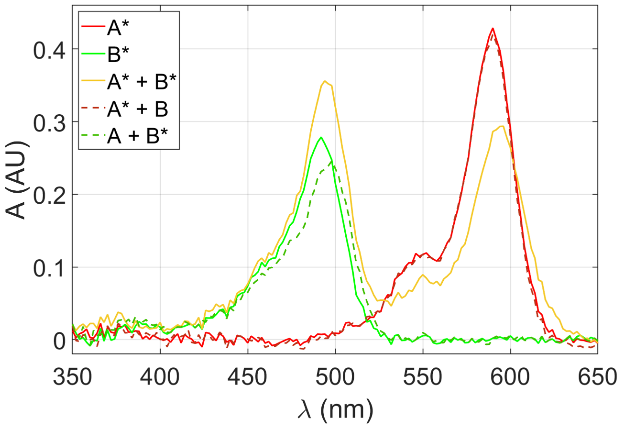

Fig F. Absorbance Spectra of labeled DNA systems: A* (red line), B* (green line), A*B* (yellow line), A*B (dark-red dashed-line) and AB* (dark-green dashed-line)

with Contact-Mediated Quenching of the Fluorescence, of which various examples involving fluorophores based on rhodamine are reported in literature [4].

VI. CONTACT-QUENCHING DATA ANALYSIS

An example of the raw fluorescence signal of a CQ experiment performed with a qPCR machine is shown in Fig Ha where we plot the fluorescence intensity I_f obtained by measuring 5 replicas - in 5 different wells - of the same sample, and for each 2 cooling ramps. While the two ramps are, for each replica, similar, the amplitude of the collected signal markedly differs between replicas. This might be due to different sensibilities of the

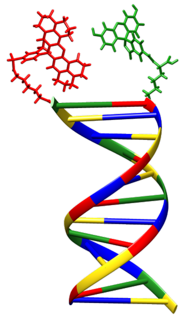

Fig G. Simplified representation of the 12A*B* duplex, showing the relative size of DNA duplex, linker and fluorescent moieties FAM (in green) and TexasRed (in red)

instrument in the distinct wells and to fluctuations in the volumes of each sample drop. However, upon normalizing all the traces to their values at high T (using the T range $60°C$-$70°C$), the curves of normalized fluorescence intensity (N_f) nicely collapse, as shown in Fig Hb . The difference between normalized curves enables evaluating the standard deviation of the errors on this signal. Since measurement of N_f becomes unstable above $70°C$, we excluded this region from data analysis.

Having characterized CQ from in solutions of A* and B*, we explored N_f in solution where only a fraction of the molecules has a fluorescent tag. We thus prepared mixtures with a fixed concentration of A* ($c_{A^*} = 100nM$) and a concentration of B* and B so that $c_{B^*} + c_B = c_{A^*}$. In this way, at low T all the A* are paired with either B or B*, depending on the fraction $f_{B^*} = c_{B^*}/c_{A^*}$. Fig I shows N_f for TexasRed (the fluorophore of A*) at $T = 20°C$ measured for various values of f_{B^*} ranging from 0 (A*B* duplexes only) to 1 (A*B duplexes only). We find $N_f(T = 20°C)$ to depend linearly on f_{B^*}, as expected from the summation of the fluorescent emissions of individual duplexes. This behaviour enables determining, through CQ, the fraction $\theta_{A^*B^*}$ of paired A*B* in the rsDNA competitive environment as:

$$N_f = \theta_{A^*B^*} \cdot N_f^{(p)} + [1 - \theta_{A^*B^*}] \cdot N_f^{(u)}, \tag{9}$$

where $N_f^{(p)}$ and $N_f^{(u)}$ are the reference signal for the paired and unpaired A*B*. Thus

$$\theta_{A^*B^*} = \frac{N_f - N_f^{(u)}}{N_f^{(p)} - N_f^{(u)}} \tag{10}$$

The reference signal $N_f^{(u)}$ of unpaired A*B* is taken by averaging the normalized emission of pure solutions of A* and of A*B. The discrepancy between these two signals is compatible with their standard deviation. The reference signal $N_f^{(p)}$ of paired A*B* in rsDNA is given by the

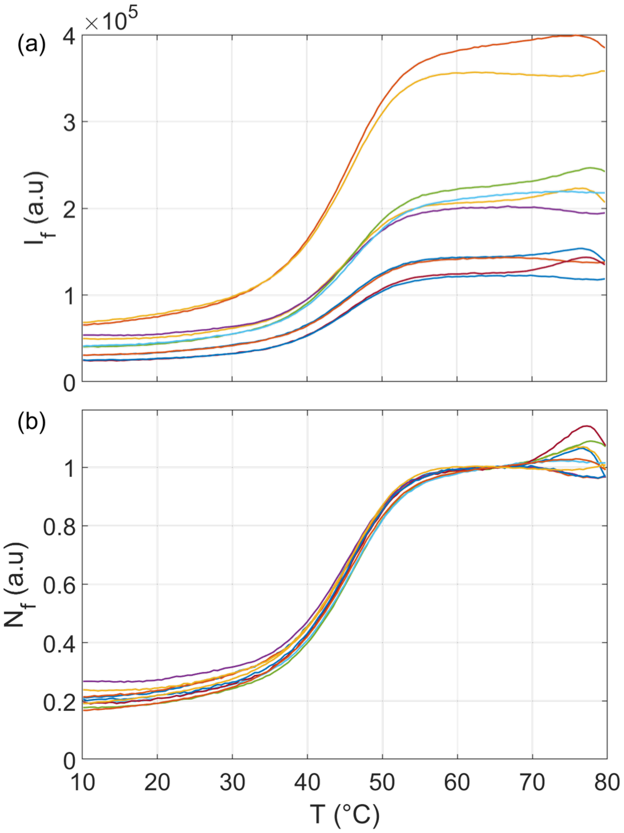

Fig H. (a): fluorescence intensity I_f of TexasRed vs. T in a solution of 12A* + 12B*, $c = 100nM$, $c_{NaCl} = 150mM$. The different curves are obtained with 2 cooling ramps of 5 replicates of the same sample. (b) normalized signal N_f of the same curves.

low T asymptote of N_f in pure solution of A*B*. This condition is not as easily determined for 8A*B* curves, where a clear saturation is not visible for a wide range of temperatures, as shown in Fig J. In this system, we fit the N_f curve using the following equation:

$$N_f(T) = \Theta(T) \cdot N_{LT}(T) + [1 - \Theta(T)] \cdot N_{HT}(T), \quad (11)$$

where $N_{LT}(T) = m_{LT}\,T + q_{LT}$ and $N_{HT}(T) = m_{HT}\,T + q_{HT}$ are the linear drifts respectively at low and high temperatures, and $\Theta_{A^*B^*}(T)$ has the functional shape expected for DNA melting curves:

$$\Theta(T) = 1 - \frac{2}{1 + \sqrt{1 + \exp\left[-\left(\frac{\mathbb{H}}{T} - \mathbb{S}\right)\right]}} \quad (12)$$

where $\mathbb{H}$ and $\mathbb{S}$ are here fitting parameters, while in the melting curve expression they depend on the enthalpic and entropic contributions to the duplex stability, on the DNA concentration and on other numerical parameters. From the fitting procedure we extract $\theta_{A^*B^*}$.

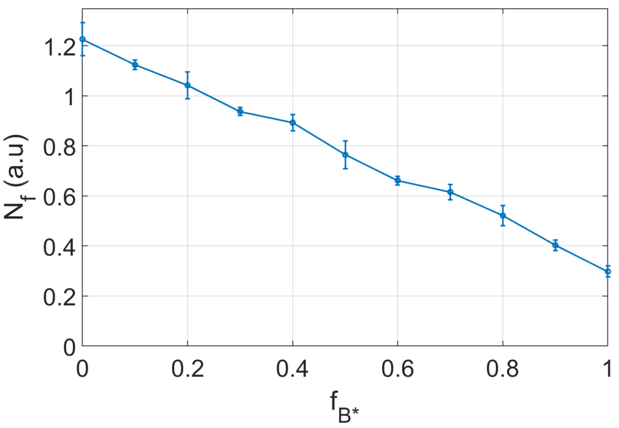

Fig I. Normalized fluorescence intensity N_f of TexasRed in a solution of 12A*, 12B* and 12B, with $c_{A^*} = 100nM$ and $c_{NaCl} = 150mM$. $c_{B^*} + c_B = c_{A^*}$. $f_{B^*} = c_{B^*}/c_{A^*}$. $T = 20°C$.

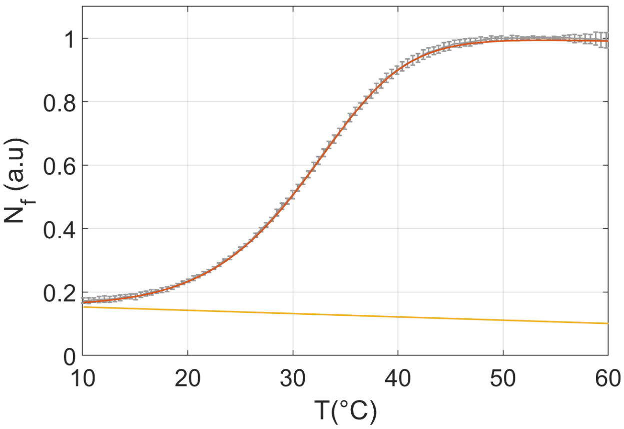

Fig J. Average normalized fluorescence N_f of 8A*+8B* (grey line). Red line: best fit using Eq. (11). The fit enables determining the linear drift at low temperatures (yellow line), corresponding to the signal of fully paired A*B*. $c_{A^*} = c_{B^*} = 100nM$. $c_{NaCl} = 150mM$

VII. FREE ENERGY OF A*B* DUPLEX

The thermodynamic stability of A*B* duplexes differs from the one of untagged AB duplexes, which can be evaluated using the Nearest Neighbor model, because of the stabilizing effect of the terminal fluorophores. Since such thermodaynmic parameters are necessary to compute the model prediction for CQ experiments, we performed a set of melting experiments at different DNA concentration. Fig K shows T_m vs concentration obtained from the experiments and the theoretical predictions from the standard Nearest Neighbor model. The stabilizing effect of the terminal fluorophores is very relevant for 8A*-8B* (red and green lines), where the difference between melting temperatures is $\Delta T_m \approx 10°C$.

We thus determined the correct ΔH and ΔS for the A*B* duplex by fitting the data with the general expression for complementary strands at the same concentration c [5]:

$$T_m = \frac{\Delta H}{\Delta S + R \cdot ln([c]/2)} - 273.15°C. \quad (13)$$

For sake of clarity, the logarithm argument is $[c]/2$ and not $[c]/4$ because here we are considering $[c] = [c_{A^*}] = [c_{B^*}]$. From the fit we have extracted the mean values of ΔH and ΔS, the standard deviations and the covariances. The T_m resulting from the fit and their uncertainties are shown in Fig K.

Given the significant stabilizing effect of the terminal fluorophores in A*B*, it is reasonable to assume that also any pair involving a strand A* or B* and a rsDNA oligomers will have a modified energetic contribution due to the presence of the fluorophores. To this aim, We thus assume that the pairing free energy $\Delta G_{A^*B^*}$ of A*B* differs from that of AB (ΔG_{AB}) because of the distinct contributions of the two fluorophores $\Delta G_{TexasRed}$ and ΔG_{FAM}:

$$\Delta G_{A^*B^*} = \Delta G_{AB} + \Delta G_{TexasRed} + \Delta G_{FAM} = \\ = \Delta G_{AB} + 2\Delta G_{fluo}. \quad (14)$$

We also assume the effect of the two fluorophores to be equal: $\Delta G_{TexasRed} = \Delta G_{FAM} = \Delta G_{fluo}$. In line with these assumptions, the pairing free energy of A* or B* with a rsDNA strand is influenced by the contribution of a single fluorophore:

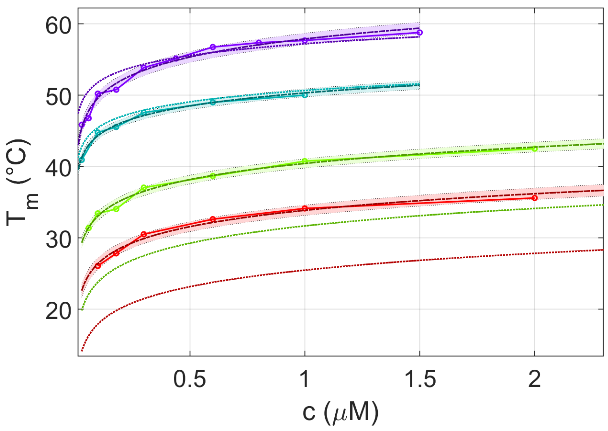

Fig K. T_m vs A*B* concentration measured by CQ for the following systems (dots): 8A*-8B* NaCl 150mM (Red), 8A*-8B* NaCl 1mM (green), 12A*-12B* NaCl 150mM (light blue), 12A*-12B* NaCl 1mM (purple). Dash-dot lines and the shaded regions are the best fit and uncertainty from Eq. (13). Dotted lines: predicted T_m from the Nearest Neighbor model using the energetic parameters for the AB duplex.

$$\Delta G_{A^*rsDNA} = \Delta G_{B^*rsDNA} = \Delta G^{(\boldsymbol{\alpha})}_{f_{CG}} + \Delta G_{fluo}, \quad (15)$$

where $\Delta G^{(\boldsymbol{\alpha})}_{f_{CG}}$ is the pairing energy computed as described in the main text, with $\boldsymbol{\alpha}$ the pairing quality of the duplex that A* or B* is forming with the rsDNA oligomer, and f_{CG} is the fraction of C or G bases of the labeled DNA strands.

1. Bellini T, Zanchetta G, Fraccia TP, Cerbino R, Tsai E, Smith GP, et al. Liquid crystal self-assembly of random-sequence DNA oligomers. Proc Natl Acad Sci. 2012;109(4):1110–1115.
2. Tataurov AV, You Y, Owczarzy R. Predicting Ultraviolet Spectrum of Single Stranded and Double Stranded Deoxyribonucleic Acids. Biophysi Chem. 2008;133:66–70.
3. Owczarzy R. Melting temperatures of nucleic acids: discrepancies in analysis. Biophys Chem. 2005; 117(3):207–215.
4. Marras SAE, Kramer FR, Tyagi S. Efficiencies of fluorescence resonance energy transfer and contact-mediated quenching in oligonucleotide probes. Nucleic Acids Res. 2002;30(21).
5. Plata CA, Marni S, Maritan A, Bellini T, Suweis S. Statistical physics of DNA hybridization. Phys Rev E. 2021;103:042503.
