# Supplementary material for: Pairing statistics and melting of random DNA oligomers: Finding your partner in superdiverse environments
Source: PLoS Comput Biol. 2022 Apr 11;18(4):e1010051. doi: 10.1371/journal.pcbi.1010051 (PMC9022813; doi:10.1371/journal.pcbi.1010051)
Supplement: S3 Text — Partition Function; Melting Curve Approximations for rsDNA solution; Energetic Parametrization based on α; Pairing Statistics with αi = 0; Effects of the Ionic Strength on the Pairing Statistics.Fig A: Melting Curves of rsDNA, according to the Low T Approximation, High T Approximation and Unification Ansatz. (PDF) [file pcbi.1010051.s003.pdf]

S3 Text. Comprehensive Description of the Theoretical Model Pairing Statistics and Melting of Random DNA Oligomers: finding your Partner in Superdiverse Environments

Here we present the development of the theoretical framework. In section I of the main text, the Partition Function is computed for a completely general system, with a set of DNA sequences, all potentially interacting with each other in multiple ways, and with each sequence present, in principle, with different concentrations. Then, starting from section II, we introduce the case of rsDNA in the thermodynamic limit. The theoretical development of the melting curve continues until Eq. (12), that is used in the main text. The parameters used in the energetic description are provided in section III. Section IV deals with the prediction of duplexes without internal mismatches in the rsDNA solution. Finally, in section V, the effect of salt concentration is discussed.

I. PARTITION FUNCTION

Let's consider S different DNA sequences, labelled from 1 to S. The i-th sequence has a total population equals to N_i identical strands. Therefore, $N = \sum_{i=1}^{S} N_i$ is the total number of oligomers. Hence, the total concentration of oligomers is $c = N/V$, whereas the number concentration of the i-th sequence is $c_i = N_i/V = \chi_i c$. The fractions of oligomers $\chi_i = N_i/N$ naturally verifies the normalization $\sum_{i=1}^{S} \chi_i = 1$.

We consider that, in principle, any duplex formation (complementary or not) can take place. Moreover, in contrast to the study of the melting curve made in Ref. [1] , we consider that two oligomers with sequences i and j can be paired in different modes $k = 1, \ldots, K_{ij}$. This leads to the definition of different global free energy changes $\Delta G_{ij}^{(k)}$ in a duplex formation comprising oligomers with sequences i and j in the k-th mode of coupling. It comes in handy to define the Boltzmann factors

$$\zeta_{ij}^{(k)} = [c] \exp\left(-\beta \Delta G_{ij}^{(k)}\right), \qquad (1)$$

where the brackets around the concentration denotes that it is measured in mol/L, and $\beta = (k_B T)^{-1}$ with k_B and T being the Boltzmannn constant and the temperature of the system respectively. Note that, when the free energy is introduced per mol, the Boltzmann constant has to be replaced by the gas constant R. In real mixtures, some duplex are very unlikely, which means that such coupling has a very high value of ΔG, or equivalently a very low value of ζ.

The hybridization state is characterized through a vector $\boldsymbol{n}$ of dimension $\sum_{i=1}^{S} \sum_{j=i}^{S} K_{ij}$ containing the number of all formed duplexes $n_{ij}^{(k)}$ comprising oligomers with sequences i and j coupled within its k-th mode. Note that we have assumed the dimension of the hybridization state neglecting repeated entries since $n_{ij}^{(k)} = n_{ji}^{(k)}$ by definition. Using the canonical distribution, as in Ref. [1], we can describe the probability of having a certain hybridization state $p(\boldsymbol{n})$ in our general system

$$p(\boldsymbol{n}) = \frac{1}{Z} f(\boldsymbol{n}) \prod_{i=1}^{S} \prod_{j=i}^{S} \prod_{k=1}^{K_{ij}} \left(\frac{\zeta_{ij}^{(k)}}{N}\right)^{n_{ij}^{(k)}}, \qquad (2)$$

with the partition function

$$Z = \sum_{\boldsymbol{n}} f(\boldsymbol{n}) \prod_{i=1}^{S} \prod_{j=i}^{S} \prod_{k=1}^{K_{ij}} \left(\frac{\zeta_{ij}^{(k)}}{N}\right)^{n_{ij}^{(k)}}. \qquad (3)$$

The sum over $\boldsymbol{n}$ is defined over all possible values of $n_{ij}^{(k)}$ that are integers and hold the set of inequalities $0 \leq 2\sum_{k=1}^{K_{ii}} n_{ii}^{(k)} + \sum_{j\neq i} \sum_{k=1}^{K_{ij}} n_{ij}^{(k)} \leq N_i$, $\forall i$. The degeneracy $f(\boldsymbol{n})$ can be explicitly written as

$$f(\boldsymbol{n}) = \prod_{i=1}^{S} \frac{N_i!}{\left(N_i - 2\sum_{k=1}^{K_{ii}} n_{ii}^{(k)} - \sum_{j\neq i} \sum_{k=1}^{K_{ij}} n_{ij}^{(k)}\right)!} \times \frac{1}{2^{\sum_{k=1}^{K_{ii}} n_{ii}^{(k)}}} \prod_{j=i}^{S} \prod_{k=1}^{K_{ij}} \frac{1}{n_{ij}^{(k)}!}. \qquad (4)$$

Above, the three groups of terms in the denominator stand for the exchangeability of the free oligomers, the oligomers inside duplex whose components share sequence, and the duplexes of all different kinds respectively.

The partition function contains all the relevant statistical information of the system. One can compute the average number of duplex comprising sequences i and j within the k-th mode taking the derivative

$$\left\langle n_{ij}^{(k)} \right\rangle = \zeta_{ij}^{(k)} \frac{\partial \ln Z}{\partial \zeta_{ij}^{(k)}}. \qquad (5)$$

If we are not interested in the modes of coupling between the duplexes, we can carry out a marginal sum over k in Eq. (2) with a straightforward application of the multinomial theorem. The result of such operation converges to the theory developed in Ref. [1] where the Boltzmann factor of each duplex is reduced to $\zeta_{ij} = \sum_{k=1}^{K_{ij}} \zeta_{ij}^{(k)}$.

The melting curve, defined as the fraction of paired oligomers in the system, is

$$\theta_e = \frac{2\sum_{i=1}^{S} \sum_{j=i}^{S} \sum_{k=1}^{K_{ij}} \left\langle n_{ij}^{(k)} \right\rangle}{N}, \qquad (6)$$

Conveniently, we can construct the global melting curve as a linear combination of individual melting curves for every single sequence in the system, that is,

$$\theta_e = \sum_{i=1}^{S} \chi_i \theta_e^{(i)} \tag{7}$$

where the individual melting curves, $\theta_e^{(i)}$, which are the fraction of paired oligomers of each species, read

$$\theta_e^{(i)} = \frac{\sum_{j=1}^{S} \sum_{k=1}^{K_{ij}} (1+\delta_{ij}) \left\langle n_{ij}^{(k)} \right\rangle}{N_i} \tag{8}$$

with δ_{ij} being the Kronecker's delta.

II. MELTING CURVE APPROXIMATIONS FOR RSDNA SOLUTION

Real experimental situations fall under the thermodynamic limit, that is, with very high number of strands in the system. Practical use of the finite size theory is unfeasible in such regime. Unfortunately, in contrast to simpler situations like those studied in Ref. [1], a clean and accurate derivation of the thermodynamic limit is not possible, since the expression of $f(\boldsymbol{n})$ denies a factorization of $p(\boldsymbol{n})$. In the following, we are going to study the thermodynamic limit using some simplifying approximations.

From now on, we consider a mixture of rsDNA oligomers of length L. Since there are four different bases, we are considering $S = 4^L$ different sequences. We assume all of them are homogeneously populated, that is, $\chi_i = 1/4^L$. Furthermore, we assume that the different modes of coupling between strands correspond to the alignment between the bases. Therefore, we consider $K_{ij} = 2L-1$ for any duplex and we replaced the index k by the integer shift parameter $-(L-1) \leq \alpha_s \leq L-1$, with α_s defined as in the main text.

A. Low T Approximation

On the one hand, let us first consider the limit of low temperatures, where the melting curve should be dominated by the ground state configuration. Therein, we can focus on the hybridization of perfectly complementary sequences with no shift ($\alpha_s = 0$). We call the complementary sequence of the i-th sequence $j^*(i)$. Self-complementary sequences satisfy $j^*(i) = i$. In such situation, all couples ij different from $ij^*(i)$ are negligible. Thus, the probability of the hybridization state factorizes for couples of complementary sequences allowing direct calculation of each individual melting curve in a simple scenario. The individual melting curve of species i reads

$$\theta_e^{(i)} = 1 - \frac{2}{1+\sqrt{1+\frac{4}{4^L}\zeta_{ij^*(i)}^{(0)}}}. \tag{9}$$

Note that, consistently, this is the melting curve obtained in Ref. [1] either for complementary couple (CC) with concentration $2\,c/4^L$ or self-complementary (SC) with concentration $c/4^L$.

B. High T Approximation

On the other hand, we consider the limit of high temperatures, where the system will be dominated by free oligomers. In this limit, we expect no significant correlations between the different duplexes and each strand can be thought of as in contact with an infinite bath. In such approximation, we get that the individual melting curve of species i is

$$\theta_e^{(i)} = 1 - \frac{1}{1+\frac{1}{4^L}\sum_{j=1}^{4^L}\sum_{\alpha_s=-L+1}^{L-1}\zeta_{ij}^{(\alpha_s)}}. \tag{10}$$

With the assumption of no correlation, the probability of occurrence of a specific pairing $ij(\alpha_s)$, assuming that i has been paired, reduces to

$$\varphi_i(j,\alpha_s) = \frac{\zeta_{ij}^{(\alpha_s)}}{\sum_{j'=1}^{4^L}\sum_{\alpha'_s=-L+1}^{L-1}\zeta_{ij'}^{(\alpha'_s)}}. \tag{11}$$

C. Unification Ansatz

A matching approximation that unifies both limits in Eq. (9) and Eq. (10) is the *Unification Ansatz*:

$$\theta_e^{(i)} = 1 - \frac{2}{1+\sqrt{1+\frac{4}{4^L}\sum_{j=1}^{4^L}\sum_{\alpha_s=-L+1}^{L-1}\zeta_{ij}^{(\alpha_s)}}}. \tag{12}$$

This expression converges to Eq. (9) for very low temperatures when the sum is dominated by the fully complementary aligned duplex; whereas for high temperatures $\zeta_{ij}^{(\alpha_s)} \ll 1$ and the square root can be expanded leading to Eq. (10). The melting curve in Eq. (12) can be interpreted as the melting curve of a self-complementary system with concentration $c/4^L$ and many modes of coupling one for each j and α_s. In Fig A we show the theoretical prediction of melting curves in the different approximations: the *Low T Approximation* (Eq. (9)), the *High T Approximation* (Eq. (10)) and the *Unification Ansatz* (Eq. (12)), in the case of a rsDNA system of length $L = 12$. Additionally, we have plotted as a reference, the melting curve for a system made of just one self-complementary couple of sequences concentrated with the same total concentration that the rsDNA solution, thus much more concentrated than the single sequences in the rsDNA system.

The *Unification Ansatz* is the most promising approximation, because it takes into account the contribution of all the kind of duplexes, differently

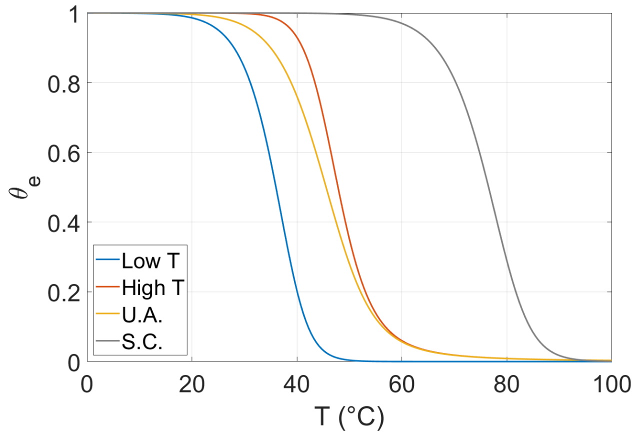

Fig A. Melting Curves of rsDNA, according to the *Low T Approximation* (blue line), *High T Approximation* (red line) and *Unification Ansatz* (yellow line); the rsDNA behaviour can be compared with the melting curve of a Self-Complementary sequence in the same experimental conditions (grey line). The curves are computed for 12N, c = 25 g/l at NaCl 1M

from the *Low T Approximation*. Moreover, if the *High T Approximation* overestimates the fraction of paired duplexes, the *Unification Ansatz* limits this inaccuracy, since its melting curve is always below.

III. ENERGETIC PARAMETRIZATION BASED ON $\boldsymbol{\alpha}$

As mentioned in section IV of the main text, we discuss here the energy parametrization on $\boldsymbol{\alpha}$ that allowed us to calculate $\Delta G^{(\boldsymbol{\alpha})}_{f_{CG}}$ associated to the DNA duplex characterized by the vector $\boldsymbol{\alpha} = (\alpha_s, \alpha_{e1}, \alpha_{e2}, \alpha_i)$, with fraction of C or G bases, f_{CG}, and length L.
The free energy difference $\Delta G^{(\boldsymbol{\alpha})}_{f_{CG}}$ between a random DNA duplex and its free random DNA sequences can be split into an enthalpic and an entropic part which also depends on $\boldsymbol{\alpha}$ and f_{CG},

$$\Delta G^{(\boldsymbol{\alpha})}_{f_{CG}} = \Delta H^{(\boldsymbol{\alpha})}(L, f_{CG}) - T\Delta S^{(\boldsymbol{\alpha})}(L, f_{CG}) \qquad (13)$$

We can describe $\Delta H^{(\boldsymbol{\alpha})}(L, f_{CG})$ and $\Delta S^{(\boldsymbol{\alpha})}(L, f_{CG})$ as a sum of the following energies components: perfect pairing, internal terminal contributions, AT terminal penalties and internal mismatches:

$$\begin{aligned}\Delta H^{(\boldsymbol{\alpha})}(L, f_{CG}) =& \Delta H_{\text{perf}}(L, f_{CG}) + \Delta H_{\text{term}}(\alpha_s, \alpha_{e1}, \alpha_{e2}) \\ &+ \Delta H_{AT-pen} + \Delta H_{\text{int}}(\alpha_i), \qquad (14a)\\ \Delta S^{(\boldsymbol{\alpha})}(L, f_{CG}) =& \Delta S_{\text{perf}}(L, f_{CG}) + \Delta S_{\text{term}}(\alpha_s, \alpha_{e1}, \alpha_{e2}) \\ &+ \Delta S_{AT-pen} + \Delta S_{\text{int}}(\alpha_i) \\ &+ \Delta S_{\text{salt}}\left(L, \boldsymbol{\alpha}, [Na^+]\right) \qquad (14b)\end{aligned}$$

Entropy is additionally corrected due to salt concentration $[Na^+]$. In the following sections, we account for each contribution in detail.

A. Perfect pairing

Let us classify bases in two categories, either strong bases or weak ones. Specifically, we consider s= $\{C, G\}$ and $w = \{A, T\}$. We firstly calculate the averaged energetic contributions [2] : ΔH^{ww}_{NN} = -7.4 kcal/mol averaged over all the possible combination of propagation sequence ww/ww, ΔH^{ss}_{NN} = -9.1 kcal/mol averaged over all the possible combination of ss/ss, and ΔH^{ws}_{NN} = -8.2 kcal/mol averaged over all the possible combination of ws/ws and sw/sw. We notice that $\Delta H^{ws}_{NN} \approx (\Delta H^{ww}_{NN} + \Delta H^{ss}_{NN})/2$; the same relation occurs for the entropic contribution, since ΔS^{ww}_{NN} = -21.07 cal/(mol K), ΔS^{ss}_{NN} = -22.85 cal/(mol K) and ΔS^{ws}_{NN}=-22.08 cal/(mol K). Consequently, we can compute the averaged enthalpy contribution of the quartets in a perfect paired duplex as the linear combination of ΔH^{ss}_{NN} and ΔH^{ww}_{NN}, weighted on f_{CG}. Analogously for the entropic contribution:

$$\Delta H_{NN}(f_{CG}) = \Delta H^{ss}_{NN} f_{CG} + \Delta H^{ww}_{NN}(1 - f_{CG}) \qquad (15a)$$
$$\Delta S_{NN}(f_{CG}) = \Delta S^{ss}_{NN} f_{CG} + \Delta S^{ww}_{NN}(1 - f_{CG}) \qquad (15b)$$

The contribution of a perfectly matched duplex is calculated as:

$$\Delta H_{\text{perf}}(L, f_{CG}) = \Delta H_0 + (L-1)\Delta H_{NN}(f_{CG}), \qquad (16a)$$
$$\Delta S_{\text{perf}}(L, f_{CG}) = \Delta S_0 + (L-1)\Delta S_{NN}(f_{CG}) \qquad (16b)$$

where ΔH_0 and ΔS_0 are initial enthalpy and entropy contribution [2].

B. Terminal Contribution

This contribution takes into account that the duplex may happen with a certain external mismatched base pairs and/or overhangs due to a shifted alignment of the DNA strands. The number of external mismatches in each end is given by α_{e1} and α_{e2} respectively, whereas the number of shifted bases is given by $|\alpha_s|$. External mismatches can be calculated as sum of two dangling bases at the same end [3] . This contributions depend on the identity of the bases. We firstly calculate the averaged contributions of a generic dangling bases $x = \{A, T, C, G\}$, next to weak bases (xw/w and wx/w) or strong bases (xs/s and sx/s); the resulting averaged contributions are ΔH^{w}_{dang} = -1.08 kcal/mol, ΔH^{s}_{dang} = -3.99 kcal/mol, ΔS^{w}_{dang} = -2.76 cal/(mol K) and ΔS^{s}_{dang} = -11.15 cal/(mol K). Analogously to the previous paragraph, we can calculate the average energy contributions for a single dangling base, weighted on f_{CG}:

$$\Delta H_{dang}(f_{CG}) = \Delta H^s_{dang} f_{CG} + \Delta H^w_{dang}(1 - f_{CG}) \tag{17a}$$

$$\Delta S_{dang}(f_{CG}) = \Delta S^s_{dang} f_{CG} + \Delta S^w_{dang}(1 - f_{CG}) \tag{17b}$$

Finally, the contribution of terminal mismatches can be parametrized as follows:

$$\Delta H_{\text{term}}(\alpha_s, \alpha_{e1}, \alpha_{e2}) = c_t(\alpha_s, \alpha_{e1}, \alpha_{e2})\, \Delta H_{dang}(f_{CG}) + - (|\alpha_s| + \alpha_{e1} + \alpha_{e2}) \Delta H_{NN}(f_{CG}) \tag{18a}$$

$$\Delta S_{\text{term}}(\alpha_s, \alpha_{e1}, \alpha_{e2}) = c_t(\alpha_s, \alpha_{e1}, \alpha_{e2})\, \Delta S_{dang}(f_{CG}) + - (|\alpha_s| + \alpha_{e1} + \alpha_{e2}) \Delta S_{NN}(f_{CG}) \tag{18b}$$

removing an energetic contribution of the perfect quarters for each unpaired bases. Note that, in order to get at least one matched base pair, we need to enforce $\alpha_{e1} + \alpha_{e2} + |\alpha_s| \leq L - 1$. Moreover, $c_t(\alpha_s, \alpha_{e1}, \alpha_{e2})$ is the parameter counting the dangling bases in the duplex:

$$c_t(\alpha_s, \alpha_{e1}, \alpha_{e2}) = \begin{cases} 0 & \text{if } \alpha_{e1} = \alpha_{e2} = \alpha_s = 0 \\ 2 & \text{if } \alpha_{e1} + \alpha_{e2} = 0 \text{ and } \alpha_s \neq 0 \\ 2 & \text{if } \alpha_{e1}\alpha_{e2} = 0 = \alpha_s \text{ and } \alpha_{e1} + \alpha_{e2} \neq 0 \\ 3 & \text{if } \alpha_{e1}\alpha_{e2} = 0 \text{ and } \alpha_{e1} + \alpha_{e2} \neq 0, \alpha_s \neq 0 \\ 4 & \text{if } \alpha_{e1}\alpha_{e2} > 0, \quad \forall \alpha_s. \end{cases} \tag{19}$$

C. Terminal AT Penalty

Here, we consider the terminal energy penalty due to AT terminal base pair. The corresponding penalty thermodynamic parameters are [2]: $\Delta H^{sing}_{AT-pen} = 2.2$ kcal/mol, $\Delta S^{sing}_{AT-pen} = 6.9$ cal/(mol K). We can calculate the terminal AT penalty weighted on f_{CG} as:

$$\Delta H_{AT-pen}(f_{CG}) = \Delta H^{sing}_{AT-pen} c_{AT}(1 - f_{CG}) \tag{20a}$$

$$\Delta S_{AT-pen}(f_{CG}) = \Delta S^{sing}_{AT-pen} c_{AT}(1 - f_{CG}) \tag{20b}$$

where c_{AT} is the parameter counting the AT penalties in the duplex:

$$c_{AT}(\alpha_s, \alpha_{e1}, \alpha_{e2}) = \begin{cases} 0 & \text{if } \alpha_s > 0 \\ 1 & \text{if } \alpha_{e1}\alpha_{e2} = 0 = \alpha_s \text{ and } \alpha_{e1} + \alpha_{e2} \neq 0 \\ 2 & \text{if } \alpha_s = \alpha_{e1} = \alpha_{e2} = 0 \end{cases} \tag{21}$$

Note that a specific well paired duplex either finish with AT or CG. The average introduced depending on the fraction f_{CG} could be seen as an strange procedure, but this is our coarse grain to model any duplex minimizing the knowledge on the specific sequence.

D. Internal Mismatches Contribution

Here, we consider the effect of internal mismatches in the duplex. The number of internal mismatches within the duplex is given by α_i. When $\alpha_i > 0$, the set of possible $\boldsymbol{\alpha}$ defining a possible duplex, with one matched base pair at least, fulfills the condition $|\alpha_s| + \alpha_{e1} + \alpha_{e2} + \alpha_i \leq L - 2$. We firstly calculate the averaged energetic contribution $\Delta H_{int}=$ 0.15 kcal/mol and $\Delta S_{int}=$ -0.84 cal/(mol K) [2], averaged over all possible propagation sequences (ww/ww, ss/ss, sw/sw, ws/ws) with only one WC pairng [4–7]. In order to prevent further complexity, we assume that additional internal mismatches are never consecutive. Therefore, the contribution due to internal mismatches is:

$$\Delta H_{\text{int}}(\alpha_i) = 2\alpha_i[\Delta H_{int} - \Delta H_{NN}(f_{CG})] \tag{22a}$$

$$\Delta S_{\text{int}}(\alpha_i) = 2\alpha_i[\Delta S_{int} - \Delta S_{NN}(f_{CG})], \tag{22b}$$

where we add and remove the energetic contribution of 2 internal mismatches and 2 perfect quartets, respectively, for each unpaired internal bases. Although we have carried out a rather arbitrary evaluation of the energetic contribution for $\alpha_i > 1$, in Fig. 6 of the main text the fraction of duplexes with even a single internal mismatch is negligible. Consequently, we expect that this evaluation doesn't affect the pairing statistics.

E. Salt Contribution

The salt contribution ΔS_{salt} to the pairing entropy ΔS has been calculated from empirical expression obtained by Owczarzy et al. [8]. Therein, we find the T_m correction described by Eq. 22, as follows:

$$\frac{1}{T_m(2)} = \frac{1}{T_m(1)} + (4.29 f_{CG} - 3.95) \cdot 10^{-5} \ln[Na^+]_2 + + 9.4 \cdot 10^{-6} \ln^2[Na^+]_2, \tag{23}$$

where $T_m(1)$ is the melting temperature at sodium reference concentration $[Na^+]_1 = 1$M, whereas $T_m(2)$ is the melting temperature at sodium ion concentration $[Na^+]_2$. Assuming that the melting of the duplexes measured in literature is due to a 2 state hybridization model, we can describe the T_m depending on the enthalpic and entropic parameters of the pairing (Eq. 19 in [1]):

$$\frac{1}{T_m(1)} = \frac{R \cdot ln(\gamma[c]) + \Delta S[1M]}{\Delta H}, \tag{24a}$$

$$\frac{1}{T_m(2)} = \frac{R \cdot ln(\gamma[c]) + \Delta S[1M] + \Delta S_{salt}([Na^+]_2)}{\Delta H}, \tag{24b}$$

where $[c]$ is the total strand concentration, γ is a multiplicative constant that depends on the considered system (self-complementary or couple of complementary strands).

From the system of equations, formed by Eq. (23), Eq. (24)a and Eq. (24)b, it is possible to obtain the salt contribution:

$$\Delta S_{salt}([Na^+]_2) = \Delta H \cdot (4.29 f_{CG} - 3.95) \cdot 10^{-5} \ln[Na^+]_2 + + 9.4 \cdot 10^{-6} \ln^2[Na^+]_2 \tag{25}$$

where ΔH can be calculated according to Eq. (14)a in our rsDNA duplexes.

IV. PAIRING STATISTICS WITH $\alpha_i = 0$

Here we discuss the pairing statistics of rsDNA in the limit of negligible internal mismatches, i.e. where all the Boltzmann weight $\zeta^{(\boldsymbol{\alpha})} \approx 0$ if $\alpha_i > 0$, compared to the statistical weights of the other pairings. As shown in Fig. 6 of the main text, this condition occurs at low temperatures, where the most probable pairings are almost perfect, i.e. characterized by a low total number of unpaired bases $|\boldsymbol{\alpha}| \equiv |\alpha_s| + \alpha_{e1} + \alpha_{e2} + \alpha_i \lesssim 2$, for the considered values of L.

In order to lighten the notation, here we neglect the f_{CG} dependence, that does not play a role in this topic. Let us rewrite the ratio of the statistical weights in this limit of negligible pairings internal mismatches:

$$\varphi_{\boldsymbol{\alpha}} = \frac{g(L,\boldsymbol{\alpha})\zeta^{(\boldsymbol{\alpha})}}{\sum_{\boldsymbol{\alpha}'} g(L,\boldsymbol{\alpha}')\zeta^{(\boldsymbol{\alpha}')}} = = \frac{g(\alpha_s,\alpha_{e1},\alpha_{e2},0)\zeta^{(\alpha_s,\alpha_{e1},\alpha_{e2},0)}}{\sum_{(\alpha'_s,\alpha'_{e1},\alpha'_{e2},0)} g(\alpha'_s,\alpha'_{e1},\alpha'_{e2},0)\zeta^{(\alpha'_s,\alpha'_{e1},\alpha'_{e2},0)}}. \tag{26}$$

Firstly, we point out that the degeneracy $g(L,\boldsymbol{\alpha})$ is L-independent if $\alpha_i = 0$ in the considered pairing, because the binomial coefficient is always 1 (see the Eq. (5) defining $g(L,\boldsymbol{\alpha})$ in the main text). Secondly, let us focus on the energetic contribution; the free energy of a pairing with $\alpha_i = 0$ can be computed as follows:

$$\Delta G(L,\boldsymbol{\alpha}) = \Delta G_0 + (L - 1 - |\alpha_s| - \alpha_{e1} - \alpha_{e2})\Delta G_{NN} + + c_t(\alpha_s,\alpha_{e1},\alpha_{e2})\Delta G_{dang} + \Delta G_{AT-pen} = = L \cdot \Delta G_{NN} + \Delta\widetilde{G}(\alpha_s,\alpha_{e1},\alpha_{e2}), \tag{27}$$

where we have separated the free energy in a L-dependent contribution $L \cdot \Delta G_{NN}$ and in a L-independent contribution $\Delta\widetilde{G}(\alpha_s,\alpha_{e1},\alpha_{e2}) \equiv \Delta G_0 - (1 + |\alpha_s| + \alpha_{e1} + \alpha_{e2})\Delta G_{NN} + c_t(\alpha_s,\alpha_{e1},\alpha_{e2})\Delta G_{dang} + \Delta G_{AT-pen}$, that takes into account only the quality of the terminals. Consequently, we can rewrite Eq. (26) distinguishing the two energetic terms:

$$\varphi_{\boldsymbol{\alpha}} = \frac{g(\boldsymbol{\alpha})[c]e^{-\beta[L\Delta G_{NN} + \Delta\widetilde{G}\alpha_s,\alpha_{e1},\alpha_{e2})]}}{\sum_{\boldsymbol{\alpha}'} g(\boldsymbol{\alpha}')[c]e^{-\beta[L\Delta G_{NN} + \Delta\widetilde{G}(\alpha'_s,\alpha'_{e1},\alpha'_{e2})]}} = = \frac{\cancel{e^{-\beta L\Delta G_{NN}}}\, g(\boldsymbol{\alpha})e^{-\beta\Delta\widetilde{G}(\alpha_s,\alpha_{e1},\alpha_{e2})}}{\cancel{e^{-\beta L\Delta G_{NN}}} \sum_{\boldsymbol{\alpha}'} g(\boldsymbol{\alpha}')e^{-\beta\Delta\widetilde{G}(\alpha'_s,\alpha'_{e1},\alpha'_{e2})}}, \tag{28}$$

where we taken the L-dependent exponential factor out of the summation, because it does not depend on $\boldsymbol{\alpha}'$. The only quantity in the equation that still depends on L is the summation $\sum_{\boldsymbol{\alpha}'}$, which is computed with the constraint that $|\alpha_s| + \alpha_{e1} + \alpha_{e2} \leq L - 1$; but, as described before, the only significant statistical weights are those of the pairings with $|\boldsymbol{\alpha}| \ll L$, so that the summation contribution with high values of $|\boldsymbol{\alpha}|$ doesn't play a role.

Thus, we have shown that the ratio of the statistical weights $\varphi_{\boldsymbol{\alpha}}$ is L-independent, in the limit of negligible pairings with $\alpha_i > 0$. In Fig. 6 of the main text it is shown the pairing statistics considering both the ratio between the statistical weights and the ensemble melting: $\theta_{\boldsymbol{\alpha}} = \varphi_{\boldsymbol{\alpha}} \cdot \theta_e$; the ensemble melting curve strongly depends on the length of the rsDNA system, but at low temperatures, i.e. $\theta_e \approx 1$ for the entire set of considered systems, also $\theta_{\boldsymbol{\alpha}}$ becomes L-independent.

V. EFFECTS OF THE IONIC STRENGTH ON THE PAIRING STATISTICS

Similarly to the previous section, we show that the pairing statistics does not depend on the salt concentration at low temperatures, as mentioned in in the discussion section of the main text. In order to lighten the notation, here we neglect the f_{CG} dependence again, that does not play any role in this topic. In Eq. (25), we notice that the logarithmic dependence of ΔS_{salt} on $[Na^+]$ as a constant factor and another proportional to ΔH of the duplex. Since at low temperature the number of unpaired bases is low, we can approximate the contribution of the salt to the entropy of in the following way:

$$\Delta S_{salt}([Na^+]) = aL\ln[Na^+] + b\ln^2[Na^+] \tag{29}$$

where a and b are constants and we have approximated $\Delta H = (L - 1 - |\boldsymbol{\alpha}|)\Delta H_{NN} \approx (L-1)\Delta H_{NN}$, since at low temperature only duplexes with $|\boldsymbol{\alpha}| = |\alpha_s| + \alpha_{e1} + \alpha_{e2} + \alpha_i \leq 2$ are significant in the pairing statistics. Consequently, we can write $\Delta G(L,\boldsymbol{\alpha}) = \Delta G'(L,\boldsymbol{\alpha}) - T\Delta S_{salt}([Na^+])$, distinguishing the salt-independet contribution $\Delta G'(L,\boldsymbol{\alpha})$ and the salt dependent $\Delta S_{salt}([Na^+])$ contribution to the free energy of the pairing. Thus, at low temperatures where $\theta_e \approx 1$, the pairing statistics can be described in the following way:

$$\theta_{\boldsymbol{\alpha}} = \frac{g(\boldsymbol{\alpha})[c]e^{-\beta[\Delta G'(L,\boldsymbol{\alpha}) - T\Delta S_{salt}([Na^+])]}}{\sum_{\boldsymbol{\alpha}'} g(\boldsymbol{\alpha}')[c]e^{-\beta[\Delta G'(L,\boldsymbol{\alpha}) - T\Delta S_{salt}([Na^+])]}} = = \frac{\cancel{e^{+\beta T\Delta S_{salt}([Na^+])}}\, g(\boldsymbol{\alpha})e^{-\beta\Delta G'(L,\boldsymbol{\alpha})}}{\cancel{e^{+\beta T\Delta S_{salt}([Na^+])}} \sum_{\boldsymbol{\alpha}'} g(\boldsymbol{\alpha}')e^{-\beta\Delta G'(L,\boldsymbol{\alpha})}}, \tag{30}$$

that does not depend on the salt concentration.

1. Plata CA, Marni S, Maritan A, Bellini T, Suweis S. Statistical physics of DNA hybridization. Phys Rev E. 2021;103:042503.
2. SantaLucia Jr J, Hicks D. The thermodynamics of DNA structural motifs. Annu Rev Biophys Biomol Struct. 2004;33:415–440.
3. Nupack;. `http://www.nupack.org/partition/new`.
4. Allawi HT, SantaLucia J. Nearest-Neighbor Thermodynamics of Internal A-C Mismatches in DNA: Sequence Dependence and pH Effects. Biochemistry. 1998;37(26):9435–9444.
5. Allawi HT, SantaLucia Jr J. Thermodynamics of internal C-T mismatches in DNA. Nucleic acids res. 1998; 26(11):2694–2701.
6. Allawi HT, SantaLucia J. Nearest Neighbor Thermodynamic Parameters for Internal G-A Mismatches in DNA. Biochemistry. 1998;37(8):2170–2179.
7. Allawi HT, SantaLucia J. Thermodynamics and NMR of internal G-T mismatches in DNA. Biochemistry. 1997;36(34):10581–10594.
8. Owczarzy R, You Y, Moreira BG, Manthey JA, Huang L, Behlke MA, et al. Effects of sodium ions on DNA duplex oligomers: improved predictions of melting temperatures. Biochemistry. 2004;43(12):3537–3554.
