# Supplementary material for: Pairing statistics and melting of random DNA oligomers: Finding your partner in superdiverse environments
Source: PLoS Comput Biol. 2022 Apr 11;18(4):e1010051. doi: 10.1371/journal.pcbi.1010051 (PMC9022813; doi:10.1371/journal.pcbi.1010051)
Supplement: Attachment — Submitted filename: response to reviewers PLOS CB.pdf [file pcbi.1010051.s004.pdf]

We thank the reviewers for their work and for their valuable suggestions, which guided us to modify the manuscript inserting clarifications and comments. We also added a figure in the supplementary information. We are confident that our manuscript is now adequate for being published.

Reviewer #1: *The authors present a combined experimental and theoretical thermodynamic study of a solution consisting of all possible L-nucleotide-long DNA oligomers (L being 8, 12 and 20) generated by the random synthesis from a mixture of equal proportion of four nucleotide precursors. Authors' main conclusion is that, despite the presence of a number of chains that can form mismatched duplexes with the given chain, which could prevent it from finding the complementary partner, the yield of fully matched duplexes at low enough temperatures is pretty high.*

Major comments

1. *The authors failed to convince this reviewer that a pretty artificial system they consider deserves so much attention. In Introduction, they claim that the system they consider is relevant to the pre-biological evolution consideration within the framework of the RNA World hypothesis. But this claim looks too far-fetched to me.*

All origin of life scenarios that share the RNA world hypothesis cannot but assume that the RNA oligomers forming the ribozymes, i.e. the first organic catalytic molecules, were the outcome of conditions favoring the random polymerization of pre-existing units. Accordingly, the RNA world must have emerged because, out of a host of randomly polymorphic oligomers, a few "lucky" sequences could fold and become active on the available substrates. Understanding whether this occurred in a system that could hybridize despite its randomness or no gives a sense of how strong a competition there was between the formation of the secondary structure of the ribozymes and the formation of bimolecular pairs, thus gauging the structural strength that the first ribozymes had to have.
In the previous version of the manuscript , the reference to the RNA world and related scenarios was brief, probably not developed enough. We expanded that comment by adding a sentence in p.3.to clarify our point. However, given the current type of publication, we prefer to keep this topic in a very concise form, leaving a thorough discussion for a future publication in the context of the prebiotic chemistry community.

2. *There is no doubt that our current understanding of thermodynamic parameters of the DNA double helix and mismatched pairs is pretty solid so the fact that the authors obtained a good agreement between theory and experiment for their artificial system is not surprising and adds very little, if any, to our understanding the DNA thermodynamics.*

We would like to emphasize that our approach is not focused on the thermodynamics of DNA hybridization, but on implementing it in a situation of enormous molecular diversity, which goes significantly beyond traditional usages. Indeed, the reviewer #2 express his/her surprise because of the excellent agreement between theoretical predictions and experiments without any fitting of free parameters and in spite of the number of approximations in our analytical derivation.
As we state at the beginning of p.3, the standard thermodynamic approach to evaluate hybridization is applicable to small numbers of molecular sequences. However, there are plenty of conditions of relevance for biology and technology where being able to evaluate the distribution of binding quality in a complex environment is important. For example, a tool to evaluate the probability that primers do not bind to their target sites in genomic or metagenomic PCR would require being able to implement the DNA thermodynamics within a robust statistical treatment of the multiplicity of potential pairs. In this spirit, we

found a way to simplify the conventional procedure to compute the DNA thermodynamics introducing averages, facilitated by a re-parametrization, as described in the paper.
To improve the clarity of our goal, we have introduced a paragraph in p. 7 stating that we do not mean to expand the current knowledge on single hybridization thermodynamics, but to enable its application in complex situations. We also have added a paragraph at the end of p. 11 - beginning of p.12 further detailing the limits of our approach and the reason we think it works so well in sufficiently complex mixtures

Technical comment. *Although the authors originally formulate the problem under consideration in very general terms, to perform a theoretical treatment they make a number more or less arbitrary assumptions, which allow them to solve the problem under consideration. For example, to calculate melting curves of fully matched duplexes they start with the nearest neighbor approximation, which takes into account the parameters of heterogeneous stacking obtained from melting experiments with long DNA duplexes, rather than using a simplified version of the melting theory, which considers the duplex stability as a function of only the duplex GC-content. But in the end, they de facto arrive at the simplified version anyway. They would make their reasoning more convincing and straightforward if they relied on a finding by Vologodskii and Frank-Kamenetskii (Phys Life Rev 25, 1-21, 2018) that theoretical models, which take into account heterogeneous stacking, do not yield better predictions of oligonucleotide duplex melting temperatures than the simplest model, which relies only on the GC-content*

We thank the reviewer for pointing out the reference, which we have included in our citations (current Ref. [11]) and to which we now refer a few times.
We considered the point he/she raises, in reference to which we would like to clarify two aspects, a fundamental one, and a practical one.
In our approach, taking into account the CG fraction is not a simplification, but an intermediate step. In fact, instead of computing straightaway a single averaged free energy and the resulting melting curve, we first consider an average of melting curves each one associated to a free energy depending on the CG content, to later average over the CG fraction.
Regarding the practical argument, the averaging performed at fixed CG content over the variety of defect statistics, has a very relevant effect. This can be appreciated Fig. S1 of SI3, where the melting expected allowing only perfect pairing in a rsDNA with fixed CG content (in this case f_CG = 0.5 – blue line), is compared with the melting expected when the contribution of the defected pairing is also considered (yellow line). The difference in both melting T and shape of the melting curve is apparent. Hence, simplifying the problem to consider only the CG content is not an adequate approximation. Indeed, the non-trivial T dependence of the statistical distribution over the defects in Fig. 6 of the main text, are all dependent on the features of the statistical summation at constant CG content.
The reviewer comment made us better aware of the necessity of better expressing this crucial element of our approach, which we do by a new portion of text added to the manuscript in p.9.

Reviewer #2: *This is a very well-done paper dealing with the ability of complementary DNA strands to find each other among a plethora of other strands, from completely incompatible to partially compatible, and taking into account also imperfect structural pairing.*
Experiments and theory nicely go hand-in-hand, and there its much to say, all in all.
Nonetheless, I want to probe the authors in two directions:

1) the theory works too well, given many approximations. It would be good if the authors could add a section for limitations of the analytical model, and where it could go wrong.

This would provide a better view for potential users of the framework about when and where this treatment is appropriate and when they should instead be careful

We thank the reviewer for this suggestion. We already had a comment about the surprising efficacy of our model at the end of p. 11, where we say that our averaging is probably made so effective by the fact that the system itself provides all possible pairing motifs. Should the system be less statistically complete, our approach would probably be less effective.
For the sake of clarity, we have completed that statement, by adding a further note on the limitations of our approach, which works at equilibrium and lacks pairing correlations. Indeed, our approach then does not provide a satisfactory model comprising competitive binding, which would be necessary when stoichiometric constraints are present.

2) Could the authors think about specific mixtures of DNA sequences designed in such a way to maximise complementarity. For example, what about ensuring a certain minimal Hamming distance (or the like) between sequences to reduce mismatches?

The question raised by the referee is quite interesting. However, while the fundamental assumption of randomness in our treatment (all sequences are equiprobable) is easily definable, the design of a mixture fulfilling the Hamming distance requirement could be extremely involved. Let us imagine that we start by a system made by two complementary sequences that we label by A and B. If we choose to add a sequence C of a given distance to A, it is not clear which is the distance between C and B. The casuistic immediately becomes tremendous.

In any case, this comment is instead related to the evaluation that we did in the SI 1 Further Results, fig. S4, where we computed what level of perfect pairing is obtained when we consider a strand at various CG content. At the limit of $f_{CG} = 1$, where the involved free energies are the strongest among the natural nucleic acids, the level of perfect pairing is larger, but still very limited, reaching 25% at most. In the spirit of the request from the reviewer, looking for a condition of reduced randomness which would increment perfect complementarity, we added a new evaluation, and namely what would happen if one were to consider nucleic acids with only two bases, with energies corresponding to the averages between CG and AT. The result is shown in the new Fig. S5, in the SI 1. When only two bases are present, the number of sequences which could pair with a given level of defects is less than in the case of the four bases, a condition favoring perfect pairing. Still, even in this situation, the perfect pairing does not exceed 35% at most.
These results, indicating how difficult is to beat randomness by strengthening WC pairing, are commented on p. 12.

Reviewer #3: *The paper by Simone De Leo and co-workers present an analysis of the melting of random DNA oligomers in "Superdiverse Environments". The article is rather technical for the journal; however, it includes detailed supplementary information.*
It is well done and shows exceptional agreement between the experimental observations and the theoretical predictions.
However, a deeper discussion of the results in the context of modern DNA in living cells, implications of their findings and possible limitation or strength due to the oligomer lengths would have been more commendable for a larger scientific audience. For instance, a more extensive discussion on the PCR primer selectivity and possible errors in the amplification would be interesting in different applications.

We thank the reviewer for his/her comments and appreciation for the work. We are aware it is a bit technical, combining rather sophisticated experimental measurements based

on fluorescence with rigorous statistical approach. However, we hope that this is also a feature making the manuscript original.
We indeed have elaborated a sort of spinoff of this manuscript in which we implemented our approach, simplified where possible, to evaluate the efficacy of cognate site binding of primers in PCR and of miRNA in cellular gene regulation. We believe that, due to its complexity and significance, this offspring of our approach has enough substance to be published apart. We added a reference to this forthcoming paper at the end of the manuscript (p.13).
